# Supplementary material for: Loco-Regional Therapies in Oligometastatic Adrenocortical Carcinoma
Source: Cancers (Basel). 2022 May 31;14(11):2730. doi: 10.3390/cancers14112730 (PMC9179919; doi:10.3390/cancers14112730)
Supplement: Supplementary file 1 [file cancers-14-02730-s001.zip › cancers-1713279-supplementary.pdf]

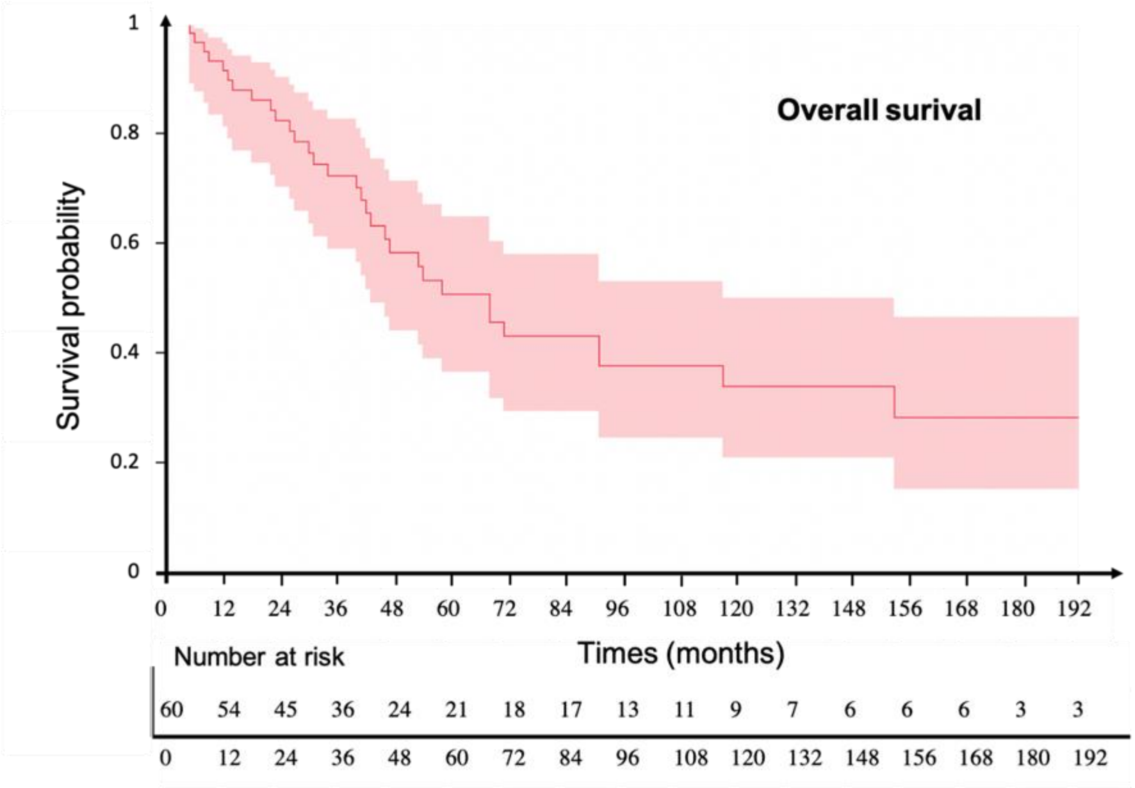

(**a**)

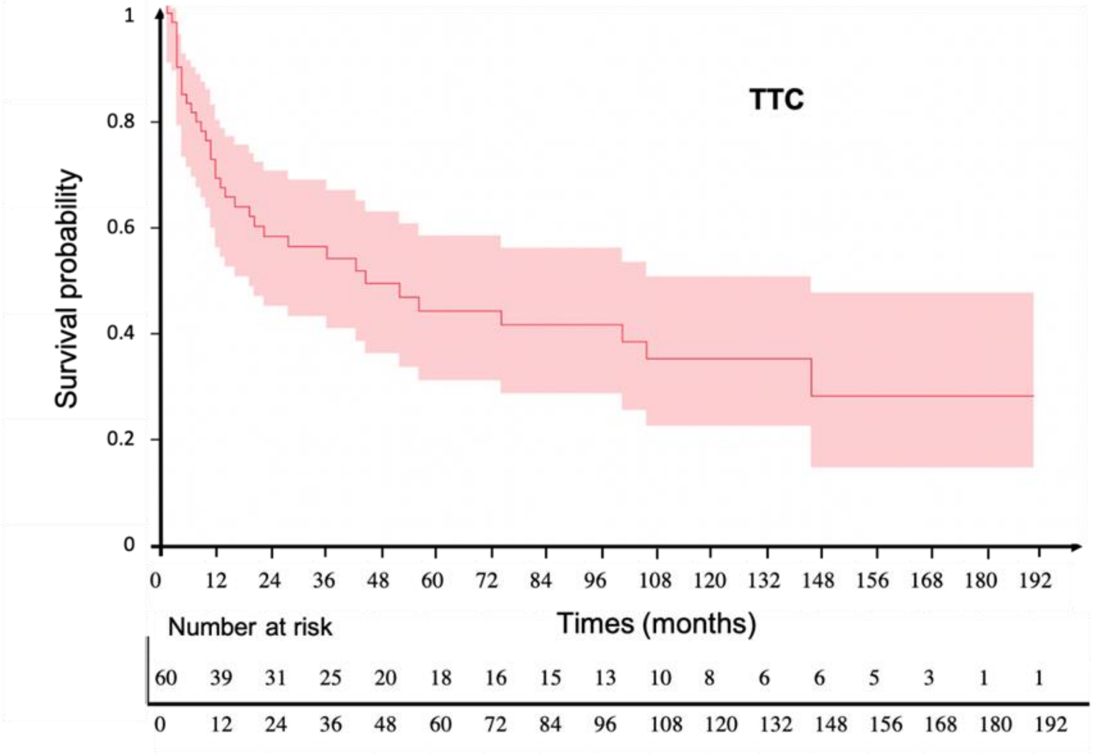

(**b**)

Figure S1. (**a**) Kaplain-meier estimate of overall survival since mitotane initiation. (**b**) Kaplain-meier estimate of time to chemotherapy since Mitotane initiation (TTC).
